# Microfluidics for the rapid detection of Escherichia coli O157:H7 using antibody-coated microspheres

**DOI:** 10.1080/21655979.2020.1870805

**Published:** 2021-01-20

**Authors:** Bo Song, Jiayuan Yu, Yan Sun, Qiao Wang, Shengnan Xu, Yichen Jia, Shuying Lin, Yueying Zhang, Chen Wang, Yingbo Zhang, Xiaojie Zhang

**Affiliations:** a Department of Clinical Pathogen, Medical Technology College, Qiqihar Medical University, Qiqihar, China; b Clinical Laboratory, Microbial Virus Group, the Second Affiliated Hospital of Heilongjiang University of Traditional Chinese Medicine, Harbin, China; c Department of Stomatology, The Second Affiliated Hospital of Qiqihar Medical University, Qiqihar, China; d Medical Technology College, Qiqihar Medical University, Qiqihar, China; e Pathology College, Qiqihar Medical University, Qiqihar, China

**Keywords:** Microfluidic chip, detection of EHEC O157: H7, ATP, microspheres

## Abstract

This study developed a novel method for the rapid detection of *Escherichia coli* (*E. coli*) O157:H7 on a microfluidic platform. First, the concentration of bacteria in a sample was determined with the adenosine triphosphate (ATP) method. Then, the specific detection of *E. coli* was achieved in a microfluidic chip by the immune-microsphere technique. The influences of the culture time, flow rate and capture time on the detection of the target bacteria were investigated systematically. Generally, with increasing capture time, more bacteria could be captured by the microspheres, which had a positive effect on bacterial detection. Furthermore, the sensitivity and specificity of the method were also tested. The results showed that this method could specifically detect *E. coli* with a sensitivity as high as 49.1 cfu/μL; the consumption of bacteria was 1 μL, and the reagent was at the microliter level. The testing time can be controlled within one and a half hours, and the cost of testing was approximately RMB 10. The method described in this article is simple and accurate and has great application value in bacterial detection for medical diagnostics.

## Introduction

*Escherichia coli* (*E. coli*) O157:H7 is a serotype of enterohemorrhagic *Escherichia coli* (EHEC) [1–3]. As the most harmful food-borne pathogenic bacterium, *E. coli* O157:H7 can destroy cells and is resistant to gastric acid. This bacterium can cause many life-treating diseases, such as hemorrhagic colitis (HC), hemolytic uremic syndrome (HUS), and thrombocytopenic purpura (TTP) [4–7]. *E. coli* O157:H7 has a strong survival ability in the external environment and can contaminate food through water and food sources [8,9]; *E. coli* O157:H7 has been widely found in meat, fruit and vegetables. The experts estimate that only 10 ~ 100 bacteria can make people sick [4]. Therefore, it is very important to detect *E. coli* O157:H7 quickly and accurately.

Many methods have been developed for detecting *E. coli* O157:H7. The gold standard culture method and serological detection method are commonly used for the clinical detection of *E. coli* [8,10]. However, the operations of these methods are tedious and include pre-enrichment, selective plating, biochemical screening, and serological confirmation [11–13]. Generally, 2 ~ 3 days are needed to complete the detection of the target bacteria. Molecular biological detection technology has also been widely used for detecting bacteria in recent decades [14,15]. As the most popular biological detection method, polymerase chain reaction (PCR) detects bacteria by amplifying a specific region of the DNA target [16,17]. With the application of PCR, the detection time decreases significantly; however, PCR detection devices are expensive, and this method is dependent on the skills of the operators. Enzyme-linked immunosorbent assay (ELISA) is a plate-based assay technique for detecting bacteria that has the advantages of fast detection speed [18], high throughput and good stability. However, the signal amplification of this method is minimal, and the choices for antibody labels are limited; therefore, the ELSA method cannot fully meet the needs of rapid detection of bacteria [13,19]

Recently, the adenosine triphosphate (ATP) luminescent method was shown to be an alternative method for cell detection. In the presence of the catalyst enzyme, ATP reacts with luciferin to emit light. Generally, the level of ATP is directly proportional to the intensity of the emitted light. By monitoring the level of ATP, the physiological and pathological properties of cells can be obtained [20,21]. In the area of bacteria detection, the ATP luminescent method can be used to measure the amount of bacteria conveniently and quickly. In comparison with previous cell counting methods [22], this method has the advantages of simple operation and quick identification, and this method may have applications in clinical detection.

Microfluidic chips have been developed for several decades [23,24]. Currently, this technology has been widely used in the fields of clinical blood, urinary tract infection (UTI) diagnosis, nucleic acid separation and quantitative analysis, immunology protein detection and cancer biology [25–28]. For example, in a recent microfluidic study, Li et al. [29] established a self-contained microfluidic chip for detecting multiplex bacteria. With this microfluidic chip, bacteria can be detected sensitively and quickly; in addition, the process is fully automated and can accelerate the diagnosis of urinary tract infections. A microfluidic system containing dendrimers and aptamers was developed to detect *E. coli* O157:H7. It significantly increased the number of aptamers on the surface of the microfluidic channel that could be used to capture *E. coli* O157:H7, for which the lower limit of detection is 10^2^ cells·mL^−1^. This system provides an effective way to develop a sensitive and rapid detection platform [30,31].

In this paper, we established a thorough microfluidic technology platform in which immunomicrospheres coated with antibodies filled the detection cavity of a microfluidic chip for bacterial detection. In order to detect *E. coli* O157:H7 rapidly, the ATP method was used to detect the total number of bacteria, and microfluidic technology with microsphere technology was used to identify the category of the bacteria. The method presented in this manuscript has the advantages of convenience and simplicity and shows great potential in developing real-time detection devices for the automatic detection of *E. coli* O157:H7.

## Materials and reagents

*E. coli* O157:H7 (GIM 1.707), *Enterococcus faecalis* (GIM 1.389) and *Vibrio cholerae* (GIM 1.449) were purchased from Guangdong microbial species preservation Center (Guangdon, China) and cultured in Lysogeny broth (LB) medium at 37°C for 24 h. Poly (dimethylsiloxane) (PDMS, Sylgard 184), and negative photoresist (SU-8 2005 Microchem) were purchased from Dow Corning Materials (USA). Anti-E. coli O157 antibody and Anti-E. coli O157antibody (FITC) were purchased from abcam company (USA), 3-aminopropyltriethoxysilane (APTES), N-hydroxysuccinimide(NHS), (1-(3-dimethylaminopropyl)-3-ethylcarbodiimide hydrochloride) (EDC) and morpholine ethanesulfonic acid MES were purchased from sigma (USA). These reagents and materials were used without further purification. ATP assay detection kit and acridine orange (AO) were purchased from Beyotime (China) and Chinese medicine group chemical reagent Co., Ltd., respectively. The glass microspheres with the diameter ranging from 50 μm to 90 μm were purchased from Sichuan Mianzhu biology company.

GraphPad Prism 8 was used to statistically calculate the results and plotted figures. The results of this experiment were expressed as (mean±standard deviation). *T*-test for pairwise comparison of normally distributed data, non-parametric test for non-normally distributed data, Pearson correlation coefficient for correlation analysis between measurement data. P < 0.050 indicates a statistically significant difference.

## Methods

### Chip design and fabrication

**Figure 1.**
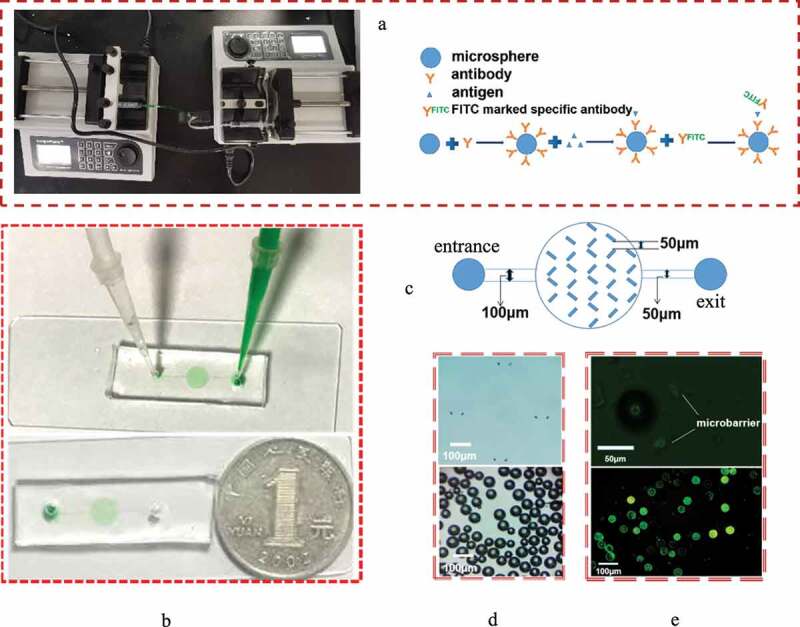
Construction of the microfluidic chip. (a) The picture of microfluidic chip connected with micro injection pump and the schematic diagram of Immunomicrosphere detection method were drawn. (b) Material object of the microfluidic chip and the comparison between the size of the prepared microfluidic chip and a single coin. The inspection hole is dyed red after adding red dye to the sample hole. (c) Design drawing of the chip. The width of two adjacent microbarriers is 50 μm to ensure that the microspheres can be captured by the microbarriers. (d) Microbarriers and microspheres under an optical microscope (magnification 10 × 40). (e) Microbarriers and microspheres under a fluorescence microscope. The diameter of the glass microspheres is 50 ~ 90 μm. The width between a pair of microbarriers is 50 µm (10 × 100)

The microfluidic chip is fabricated by combining the top poly(dimethyl siloxane) (PDMS) layer to a glass slide. The PDMS layer is fabricated by the traditional soft lithography method and contains the structure of the microchannel. In brief, photoresist is spin-coated onto a silicon wafer, soft baked, exposed, baked postexposure and developed; then, a master with a raised designed pattern is prepared. After this, a degassed PDMS-curing agent mixture is added onto the surface of the master. By placing the master into an oven and heating at 80°C for 100 min, a solid PDMS layer containing a microchannel structure can be formed. Finally, the microfluidic chip can be formed by bonding the PDMS layer and the substrate together via plasma treatment (PDC-32, Harrick) (Figure 1 (a, b)).

The microfluidic channel consists of a main entrance channel with a width of 100 µm, a culture chamber and a main exit channel with a width of 50 μm (Figure 1(c)). For anchoring the microspheres, the array of V-shaped microbarriers is placed uniformly in the microchamber (Figure 1(d, e)). The width of the large end of the V-shaped microbarriers is 50 µm, which is slightly smaller than the diameter of the microspheres. The microchannel structure has a uniform height of 100 µm.

### Detection of the concentration of bacterial suspension with the ATP method

The ATP detection method was calibrated by finding the relationship between the detected relative light unit (RLU) and the concentration of the bacterial suspensions. First, bacterial suspensions with different concentrations were prepared. The 96-well plate culture method was used to count bacteria to determine the concentrations of suspensions. Meanwhile, these bacterial suspensions were detected with the ATP detection method. ATP detection was conducted by bacterial cracking, mixing with luciferase and detection of the relative light unit (RLU).

### Preparation of immune microspheres

Ten milligrams of glass microspheres were weighed and soaked in piranha solution (H_2_SO_4_: H_2_O_2_ = 3:1) overnight. The microspheres were washed with distilled water 5 times, dried at 70°C for 30 min and immersed in 2% acetone solution at room temperature.

The reaction solution was prepared by mixing 50 μL MES buffer, 8 μL 4 mg·mL^−1^ EDC and 12 μL 4 mg·mL^−1^ NHS into 10 μL (0.1 mg·ml^−1^) Anti-*E. coli* O157 antibody working solution. The microspheres were reacted for 15 min, and 120 μL 0.1 mol·l^−1^ PBS buffer was added to stop the formation reaction. Then, the microspheres were suspended into the reaction solution at room temperature overnight for incubation.

### *Experimental procedure for the detection of* E. coli

In the experiment, the immune microspheres were first added into the microfluidic chip. Due to the presence of microbarriers, the glass microspheres were trapped in the microchamber. The bacterial suspension was pumped into the microfluidic chip through the inlet by using a syringe pump (LSP02-1B, Longer Precision Pump Co., Ltd.).

#### Detection of the culture performance of microfluidic chip

In order to verify whether microfluidic chips are suitable for cultivating bacteria, the traditional plate method was used as a control. The microfluidic chip was cultured for 0, 2, 4, 6, 8, 10, 12, 14, and 16 h, and the OD (Optical Density) value was measured by a Thermo Scientific Microplate Reader, with the wavelength at 600 nm.

#### Determination of the optimal injection flow rate and the best capture time

AO (5 µg·ml^−1^) was added to the *E. coli* O157:H7 solution at 0.5 MCF concentration cultured in 37°C LB medium and stained for 1 h. Bacterial samples (1 µL) were added into the immune enrichment chamber of the experimental group and the control group at flow rates of 1 µL·min^−1^, 2.5 µL·min^−1^ and 5 µL·min^−1^, respectively. Under the fluorescence microscope, the time from the beginning of sample injection to the time when the bacterial fluorescence particles completely entered the enrichment cavity was calculated.

Bacterial sample (1 µL) was added to the experimental group and control group, and the time allowed for the bacteria to attach onto the microspheres was changed from 2 min to 12 min. The experimental group was filled with antibody-modified microspheres. The control group was filled with microspheres only sealed with 1% BSA. The number of bacteria was counted with the traditional plate method. The capture rate of the experimental group chip was calculated according to Formula 1. The difference between the capture rate of the experimental group and the adsorption rate of the control group is taken as the actual capture rate. Five samples were measured in parallel.

Capture rate = (number of bacteria in original solution – number of bacteria in flushing solution)/number of bacteria in original solution × 100% (Formula 1)

#### Sensitivity test

Bacterial suspensions (1 µL) with concentration ratios of 1:1, 1:10, 1:10^2^, 1:10^3^, 1:10^4^, 1:10^5^, 1:10^6^, 1:10^7^ and 1:10^8^ were added to the microchamber containing immune microspheres. The initial concentration of the bacterial suspension was 4.91 × 10^8^ cfu/μL, according to the ATP equation between RLU and a bacterial suspension of known concentration. Anti-*E. coli* O157 antibody (FITC) diluent (1 µL) was added to the microchamber. The detection areas of the microfluidic chip were observed by fluorescence microscopy (IX73, Olympus), and the mean optical density of the observed results was analyzed by ImageJ software. Under each condition, the bacterial solution was detected three times.

#### *Verification of the specific detection of* E. coli

Three different bacteria (*E. coli* O157, *Enterococcus faecalis* and *Vibrio cholerae*) were detected by a microfluidic chip, and their fluorescence intensity was quantified. The experimental conditions were the same as in section 3.4.2.

## Results

A microfluidic chip platform was designed and fabricated to detect E. coli O157, the chip performance, sensitivity and specificity were verified, the experimental results are as follows.

### Determination of the concentration of bacteria by ATP method

**Figure 2.**
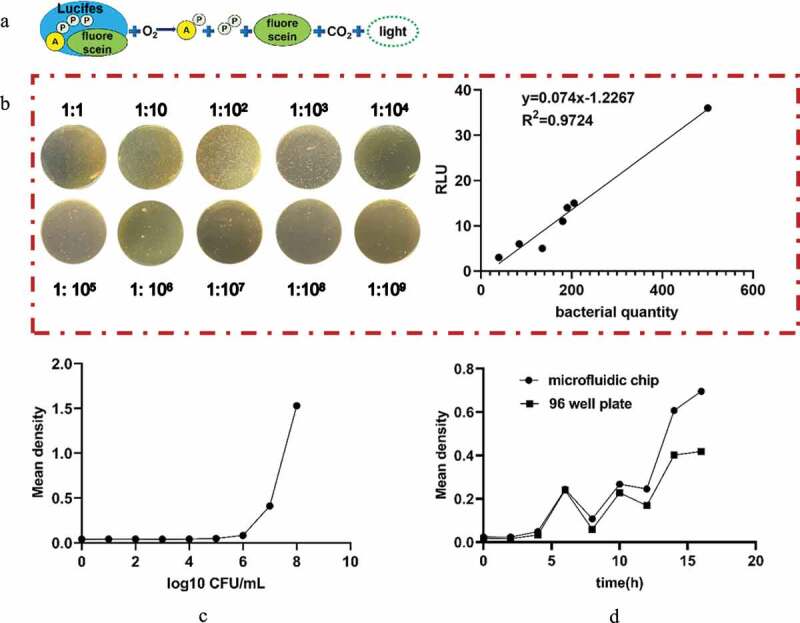
(a) Schematic diagram of the principle of the ATP detection method. (b) Relationship between the RLU and bacterial quantity. (c) Standard curve of bacterial growth of bacterial quantity. (d) Comparative observation of the growth curve of bacteria cultured by the microfluidic chip method and traditional 96-well plate method for 0, 2, 4, 6, 8, 10, 12, 14, and 16 h

The ATP method was used to determine the different concentrations of bacterial suspensions by artificial preparation (Figure 2(a)). The relationship between the RLU measured by the ATP method is linear with the concentration of the bacterial suspension (Figure 2(b)). The expression between RLU and the concentration of the bacteria is given by the following equation:

$$y=0.074x-1.2267\;R^{2}=0.9724$$

where x is the concentration of the bacterial suspension and y is the measured RLU. With this equation, the concentration of bacteria in a sample can be obtained by simply measuring the RLU with the ATP method.

### Verification of culture characteristics

The optical density (OD) value can indirectly reflect the number of bacteria. In this paper, we generated a standard curve between the concentration of the bacterial solution and the OD value (Figure 2(c)), and the concentration of the bacterial solution was calculated according to the standard curve. With the increase in capture time, more bacteria can be bonded to the microspheres. The variations in the OD values between the traditional 96-well method and the microfluidic chip method were similar when the culture time was within 16 h (Figure 2(d)). Hence, the reliability of the microfluidic chip method can be confirmed.

### Determination of the antibody injection flow rate and the best detection time

**Figure 3.**
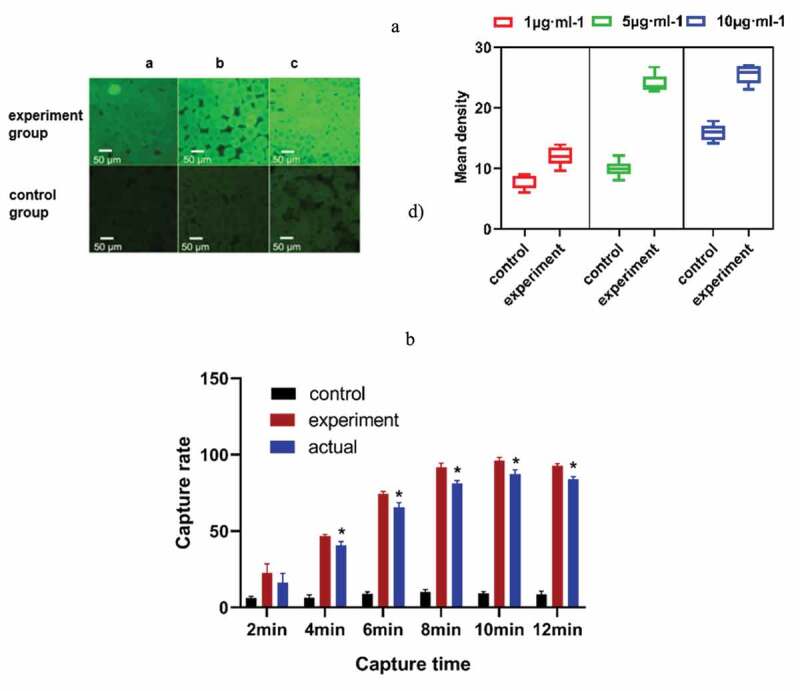
(a) The appropriate speed of sample addition was selected. The fluorescence intensity of the experimental group was obviously different from that of the control group at 5 μL·min^−1^. (b) The capture rate of the microfluidic chip at different capture times. * *P*< 0.05, Comparison with the control group. (control group, only 1% BSA-sealed glass microspheres; experimental group, antibody modification on the surface of the microspheres)

The experimental results showed that bacteria can be fully captured under different flow rates of sample addition (Figure 3(a)). In this experiment, we chose 5 μL·min^−1^ as the speed of the experiment. With the increase in time, more bacteria will be adsorbed on the microspheres. When the capture time was 10 min, the adsorption amount reached the maximum (Figure 3(b)). Considering the problem of detection accuracy and time, the best capture time was 10 min, and the highest actual capture rate of the chip was 87.27% (Table 1).

**Table 1.** Capture rate under different capture time (%, n = 5 x ± s)

Reaction time (min)	Control group	Experience group	Actual capture rate
2	0.82 ± 7.62	19.52 ± 3.01	11.92 ± 3.19
4	6.46 ± 1.77	46.66 ± 1.11	40.6 ± 2.53*
6	9.09 ± 1.05	74.29 ± 1.6	65.4 ± 3.17*
8	10.17 ± 1.49	91.69 ± 2.8	81.32 ± 1.8*
10	9.19 ± 1.14	96.052 ± 2.28	87.27 ± 2.82*
12	8.7 ± 2.16	92.74 ± 1.323	84.04 ± 1.48*

(control group: only 1% BSA sealed glass microspheres; experimental group: antibody modification on the surface of microspheres.)

* *P*< 0.05, Comparison with the control group.

### Sensitivity test

**Figure 4.**
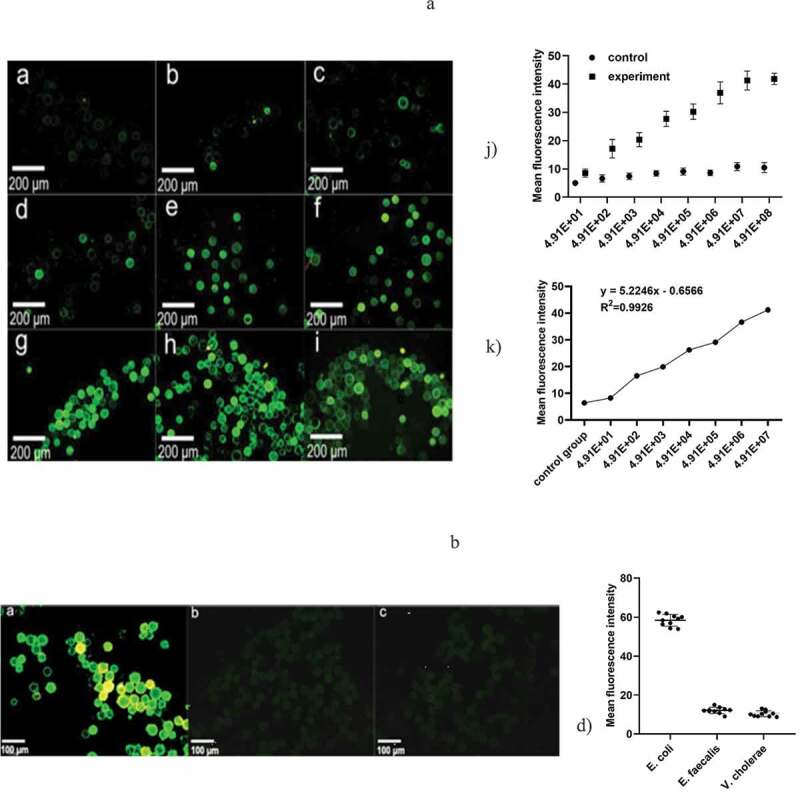
(a) Represents the negative control group, and b-i) represent the bacterial concentration in turn; 4.91 × 10, 4.91 × 10^2^, 4.91 × 10^3^, 4.91 × 10^4^, 4.91 × 10^5^, 4.91 × 10^6^, 4.91 × 10^7^, 4.91 × 10^8^ under a fluorescence microscope, respectively (magnification 10 × 40). k) The variation of the measured OD value as a function of the concentration of the *E. coli* suspension. h) Standard curve of *E. coli* at concentrations from 4.91 × 10 ~ 4.91 × 10^7^ cfu/mL and mean fluorescence intensity. (b) a)-c) Fluorescence staining results of *E. coli* O157, *Enterococcus faecalis* and *Vibrio cholerae*. d) Fluorescence mean density value of the three kinds of bacteria. (control group, only 1% BSA sealed glass microspheres; experimental group, antibody modification on the surface of the microspheres)

The control group was filled with microspheres only sealed with 1% BSA. Through comparison of the fluorescence, we could clearly see that there was no obvious fluorescence in the control group, while the fluorescence intensity in the experimental group increased with increasing bacterial concentration (Figure 4(a)). The microfluidic chip had good linearity in the concentration range of 4.91 × 10 ~ 4.91 × 10^7^ cfu/μL, which can be expressed by the regression equation

y = 5.2246x − 0.6566 (Figure. 4a(k)),

where x is the bacterial suspension, y is the mean density, and the correlation coefficient (R^2^) is 0.9926.

### Verification of microfluidic chip specificity

Fluorescent images of the microspheres were captured when different bacterial suspensions were injected into the microfluidic chip (Figure 4(b)). The fluorescence intensity of *E. coli* was much larger than that of *Enterococcus faecalis* and *Vibrio cholerae*, indicating that the microspheres emit intensive green fluorescent light only when the *E. coli* suspension was detected. Therefore, it can be demonstrated that the microfluidic chip developed in this paper has good specificity. We chose microspheres sealed with 1% BSA as the control group.

## Discussion and conclusion

In this study, a microfluidic platform based on microsphere technology was designed to culture and detect bacteria [32]. The ATP detection method was used to determine the concentration of bacteria in the samples. Generally, within 1 min, the concentration of bacteria can be measured [33]. Compared with the traditional standard method, the method developed in this study has the advantages of simple operation, fast speed, and easy on-line monitoring of microbial dynamic changes; furthermore, this method is a reliable alternative method for microbial detection (R^2^ = 0.9724 > 0.90). As the ATP method cannot conduct specific detection, the microfluidic platform combined with glass microspheres was designed to detect a category of bacteria based on antigen antibody reactions. In this study, we developed microstructures by introducing microspheres to significantly increase the surface area so that more bacteria could attach to the surfaces of microspheres, and the detection accuracy was increased. We demonstrated the ability to trap bacteria by V-shaped microbarriers to prevent the loss of microspheres. V-shaped micro barriers are set in the microchamber, and the width of the adjacent micro bracket is slightly smaller than the diameter of the microspheres.

The experimental results showed that the microfluidic chip could detect 1 μL bacteria, and the consumption of reagents was at the microliter level. The maximum actual capture rate was 87.27%, and the best capture time was 10 min while the traditional cultivation detection time is 18 to 24 h. There was a good linear relationship (R^2^ = 0.9926 > 0.90) between the average fluorescence optical density of the chip and the concentration from 4.91 × 10 to 4.91 × 10^7^ cfu/μL. Therefore, the minimum detection limit is 4.91 × 10 cfu/μL, and the maximum is 4.91 × 10^7^ cfu/μL, which is in accordance with the passive microfluidic chip trend as well [34]. The cost of the microfluidic chip designed in this experiment is approximately RMB 10, less than one tenth of the current market price. In comparison with other methods, this method has the advantages of simple chip structure, low cost and less reagent waste [35].

In this experiment, the capture time and sensitivity of captured pathogens were explored. The method improves the capture efficiency of the chip and lays the experimental foundation for the further development of high sensitivity and fast detection systems [18,36,37]. The captured efficiency of the chip was improved by exploring the time and sensitivity to captured pathogens in this experiment. A preliminary experimental foundation for the further development of high sensitivity and rapid detection systems was laid.

## Conclusion

In the chip, we used microspheres to capture bacteria, which increases the capture rate. We used V-shaped microbarriers that the width of the adjacent micro bracket is slightly smaller than the microspheres to prevent the loss of microspheres. The results show that the microfluidic platform designed in this experiment is suitable for bacterial growth. The best capture time was 10 min, and the highest actual capture rate of the chip was 87.27%. The minimum detection limit is 4.91 × 10 cfu/μL, while traditional cultivation method is 0.5 MCF. The chip used in this experiment is small and portable, while the traditional method needs large experimental equipment.

## Highlights

1. This paper developed a novel method for the rapid detection of Escherichia Coli (E. coli) O157:H7 on a microfluidic platform.

2. The testing time can be controlled within one and a half hours.

3. The cost price of testing is about RMB 10.

4. This method can conduct specific detection of E. coli with the sensitivity as high as 49.1 cfu/μL.
